# Broadband high-efficiency 3-bit coding metasurface in transmission mode based on the polarization conversion technique

**DOI:** 10.1038/s41598-023-37663-3

**Published:** 2023-07-12

**Authors:** Majid Karimipour, Mohammad Bagher Heydari, Iman Aryanian

**Affiliations:** 1 Department of Electrical Engineering, Arak University of Technology, Arak, Iran; 2 Iran University of Science and Technology, Tehran, Iran; 3 Faculty of Technical and Engineering, Imam Khomeini International University, Qazvin, Iran; 4 Iran Telecommunication Research Center (ITRC), Tehran, Iran

**Keywords:** Electrical and electronic engineering, Characterization and analytical techniques

## Abstract

The main drawback of the transmissive focusing metasurface (TFM) is its low operational bandwidth and aperture efficiency. Increasing both of these radiation characteristics simultaneously is a major challenge for these structures. This paper introduces a novel multi-state coding metasurface that utilizes system-level and element-level synthesis approaches to enhance frequency bandwidth and aperture efficiency. Unlike most of the TFMs proposed in this field, the proposed novel element consists of only two dielectric layers. The multi-frequency phase synthesis (MFPS) approach, a well-established broadband technique, is utilized for the system-level synthesis approach. An optimization algorithm is utilized to balance the phase error in the whole band in terms of gain variations and aperture efficiency. At the element design level, a PCT-based wideband technology is utilized and implemented by a subwavelength non-resonant element. The element is composed of three C-shaped metallic patterns, and the metal layers are printed on both sides of two identical dielectric layers without using any metalized via in the configuration. By simply changing the angle of arc curves in all layers, eight states of phase quantization are achieved. The amplitude of the transmitted wave with rotated polarization is larger than 0.9 from 12.3 to 16.5 GHz, except for state 4, which has an amplitude greater than 0.5 at the beginning of the band. A 25 $\times$ 25-element TFM was designed, fabricated, and tested using the aforementioned broadband technique (MFPS along with PCT-based wideband technology). The measurement results show that the 1-dB gain bandwidth of the antenna is 12.3–16.5 GHz, which is equivalent to 29%. The maximum measured aperture efficiency is 53.6%, occurring at 12.8 GHz. The proposed metasurface is classified in the group of broadband high-efficiency TFMs.

## Introduction

Combining the advantages of parabolic and phased array radiators, a new class of high-gain spatially-fed radiator is introduced in both transmitting and reflecting modes. This innovative radiator utilizes a spatial feed system to eliminate the complicated feeding network and reduce undesirable losses associated with the feeding structure^1,2^. In comparison with reflecting focusing metasurface (RFM), TFM offers a promising solution to mitigate feed blockage effects while maintaining benefits such as low profile, cost-effectiveness, ease of fabrication, and lightweight design. However, designing a highperformance TFM poses two main challenges.

The first challenge is achieving a wide working frequency bandwidth, which is inherently limited by the narrowband behavior of phasing elements. The second challenge is achieving high aperture efficiency, which is affected by the inherent insertion loss of metasurfaces in the transmissive mode. While the former challenge is common to both RFM and TFM, the latter challenge is more pronounced in TFM. This is why, unlike RFM, which can achieve high aperture efficiency even with single-layer configurations^3–5^, high-efficiency TFMs are typically implemented using multi-layer metasurfaces^6–8^. To address these challenges, various efforts have been made to enhance the bandwidth and aperture efficiency of TFMs^9–16^. Broadband approaches can generally be categorized into three groups: (i) the use of multi-layer frequency selective surfaces^1,12,13^, (ii) the utilization of sub-wavelength non-resonant unit cells and true-time-delay structures^14,15^, and (iii) the implementation of receive/transmit approaches using multi-layer configurations. The latter approach is also employed in folded TFM configurations, which require at least three metallic layers for phase and polarization manipulation^16^. Elements supporting two orthogonally polarized waves, such as V- or Y-shaped elements, exhibit broader resonances compared to classical phasing elements, offering potential improvements in bandwidth^9^.

While previous studies have focused on enhancing either the TFM bandwidth or aperture efficiency, achieving both simultaneously has received less attention. For instance, a study^9^ combined multiple broadband techniques to design an ultra-wideband configuration, but the peak aperture efficiency remained below 50% due to element loss. Similarly, another study^10^ introduced a linearly polarized TFM with a three-metallic phasing element, achieving a peak aperture efficiency of 55% but limiting the 1-dB gain bandwidth to 15.5%. Additionally, a high-efficiency TFM based on the Huygens metasurface concept was proposed^17^, attaining 64% aperture efficiency, albeit with a challenging fabrication process, and a 1-dB gain bandwidth of only 14%^17^. Similar limitations were observed in^18^. Recently, a high-gain dual-polarized bulky 3D full metal TFM was introduced using a quadruple-ridged waveguide as the unit cell^19^. This design achieved a measured peak aperture efficiency of 41.9% and a 1-dB gain bandwidth of 27.3%, representing a relatively good performance in these aspects. In reflection and transmission modes, the phase quantization scheme has been recognized as a favorable solution for metasurface design due to its simplicity and reconfigurability^20–22^. We have discovered that utilizing coding structures with the phase quantization scheme provides the best solution for achieving a high-efficiency broadband TFM. By using a set of multi-degree-of-freedom elements, we can individually adjust the dimensions of the elements to achieve the desired geometry for each bit response. The selection process considers two crucial factors: low insertion loss and the required phase value. These factors collectively ensure the broadband and high-efficiency behavior of the metasurface.

In this paper, our objective is to simultaneously address the two aforementioned factors and develop a compact broadband high-efficiency TFM using a 3-bit coding metasurface. The main novelty of our proposed work, in comparison to previous coding metasurfaces, lies in the incorporation of both broadband techniques at the element and system design levels. By integrating these techniques with a 3-bit metasurface that exhibits lower phase error than 1- and 2-bit configurations, we enhance the aperture efficiency. Additionally, the absence of metallized vias in the element’s geometry allows for its utilization at millimeter wave frequencies and beyond, minimizing errors during the construction and assembly process in multi-layer configurations. This is particularly crucial at higher frequencies, where the use of metallized vias in structures can contribute significantly to manufacturing errors^23^.

## Element design

**Figure 1:**
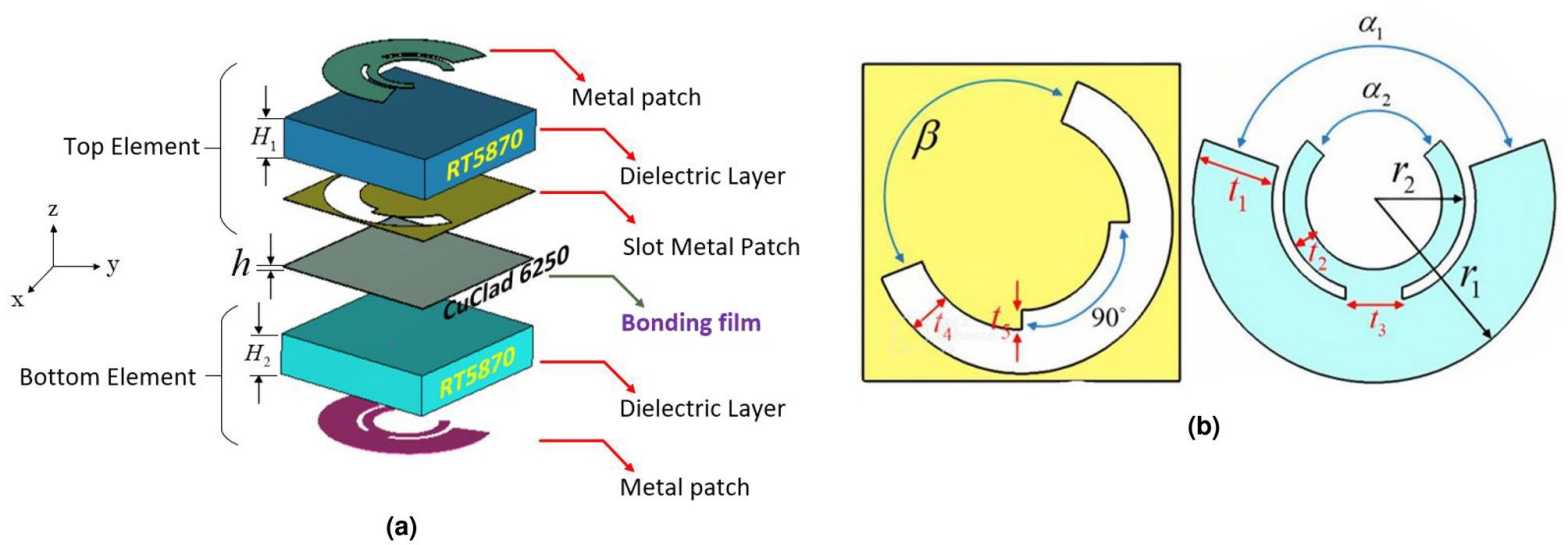
Geometry of the meta-atom. (**a**) The exploded view and (**b**) design parameters of the unit cell.

The proposed subwavelength unit cell is demonstrated in Fig. 1, which is properly designed based on multi-degree-of freedom technology such that it fulfills the phase and amplitude requirements of all eight states of the 3-bit coding metasurface. Achieving a broadband high-efficiency structure relies on using elements with multi-degree-of-freedom. These elements provide better control over the transmission coefficient of waves in terms of both amplitude and phase. An ordinary anisotropic patch (such as dog-bone unit cell) with limited control over its geometrical parameters cannot meet our expectations. This aspect is particularly important in transmissive metasurfaces compared to reflective ones, as variations in frequency have a more significant impact on the amplitude of transmitted waves in transmission mode. Therefore, it is necessary to employ a phasing element that can effectively control the electromagnetic behavior of transmitted waves across a wide frequency range. Furthermore, it is worth noting that polarization twisted surfaces offer distinct advantages over classical metasurfaces, including enhanced bandwidth and easier realization of phase states in coding metasurfaces. For instance, in a 3-bit metasurface with eight discrete phase levels, only optimizing the dimensions of four unit cells is required, while the remaining four phase states can be easily achieved by rotating the metal patch by ${180^ \circ }$ without any additional modifications to the geometrical shape of the patches. The unit cell in our design consists of three metallic layers and is based on the transmit/receive topology, without the use of any metallic vias between the layers. The top and bottom metallic patterns are double C-shaped patches with identical dimensions, connected by a narrow strip at the middle arc positions. The thicknesses of the top and bottom patches are denoted as ${{t}_{1}}$ and ${{t}_{2}}$, respectively, while the strip thickness is represented by ${{t}_{3}}$. The opening angles of the arc sections in the C-shaped patches are given as ${\alpha _1}$ and ${\alpha _2}$. The lower metallic layer of the top element is a C-shaped slot patch with slot widths of ${t_4}$ and ${t_5}$. The optimized design parameters for the unit cell can be found in Table 1.

**Figure 2:**
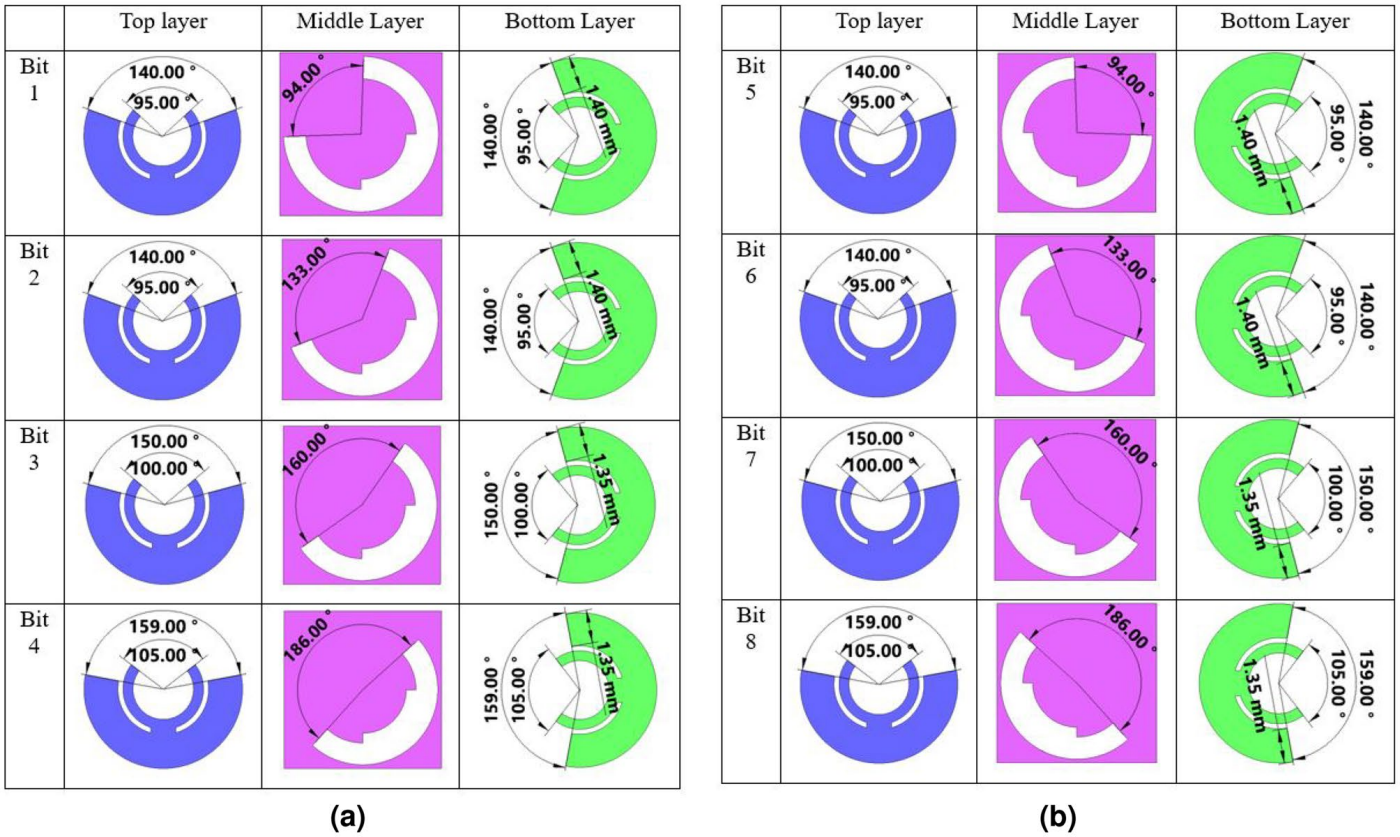
(**a**) Four states to provide transmission phases of ${130^ \circ }$, ${175^ \circ }$, ${220^ \circ }$, ${265^ \circ }$. (**b**) Four states to provide transmission phases of ${310^ \circ }$, ${355^ \circ }$, ${40^ \circ }$, ${85^ \circ }$.

The dimensions of the unit cell are set to be 6.5 mm $\times$ 6.5  mm, equivalent to $0.3{\lambda _0}$ at the center frequency of 14 GHz. The bottom and top dielectric layers are made of 1.57 mm-thick Rogers 5870 material with a relative permittivity ${\varepsilon _r} = 2.33$ and a loss tangent of 0.0012. Fig. 1 illustrates the bonding of the top and bottom elements using a 0.038 mm thick thermoplastic bonding film (Cuclad 6250). By adjusting the opening angles of the patches and slot in all three metallic layers, we are able to manipulate the transmissive phase. Thanks to the use of a sub-wavelength non-resonant structure, our element exhibits desirable behavior across a wide frequency range in terms of magnitude and phase responses. Figure 2 demonstrates the eight states of transmissive phase, covering a full cycle of ${360^ \circ }$. From Fig. 2, it is evident that the desired phases for the first four states (i.e., states of 1, 2, 3,  and 4) can be achieved by tuning the parameters ${\alpha _1}$, ${\alpha _2}$, and ${\beta }$. The remaining four states can be obtained by simultaneously rotating the middle and bottom metal patch layers by ${90^ \circ }$ and ${180^ \circ }$ around the z-axis, thanks to the polarization conversion characteristic of the element in transmission mode. Consequently, in the design of our 3-bit TFM operating in polarization conversion mode, we only need to determine four states of phases for the unit cells.

**Table 1:** Design parameter values of the proposed unit cell.

Parameter	${r_1}$	${r_2}$	${t_1}$	${t_2}$	${t_3}$	${t_4}$	${t_5}$
Value (mm)	3.2	1.6	1.4	0.4	1	0.9	0.4

Parameter	*p*	${\alpha _1}$	${\alpha _2}$	$\beta$	${H_1}$	${H_2}$	*h*
Value (mm)	6.5	8 states	8 states	8 states	1.57	1.57	0.038

## Theoretical calculation of EM response of the transmitted wave

**Figure 3:**
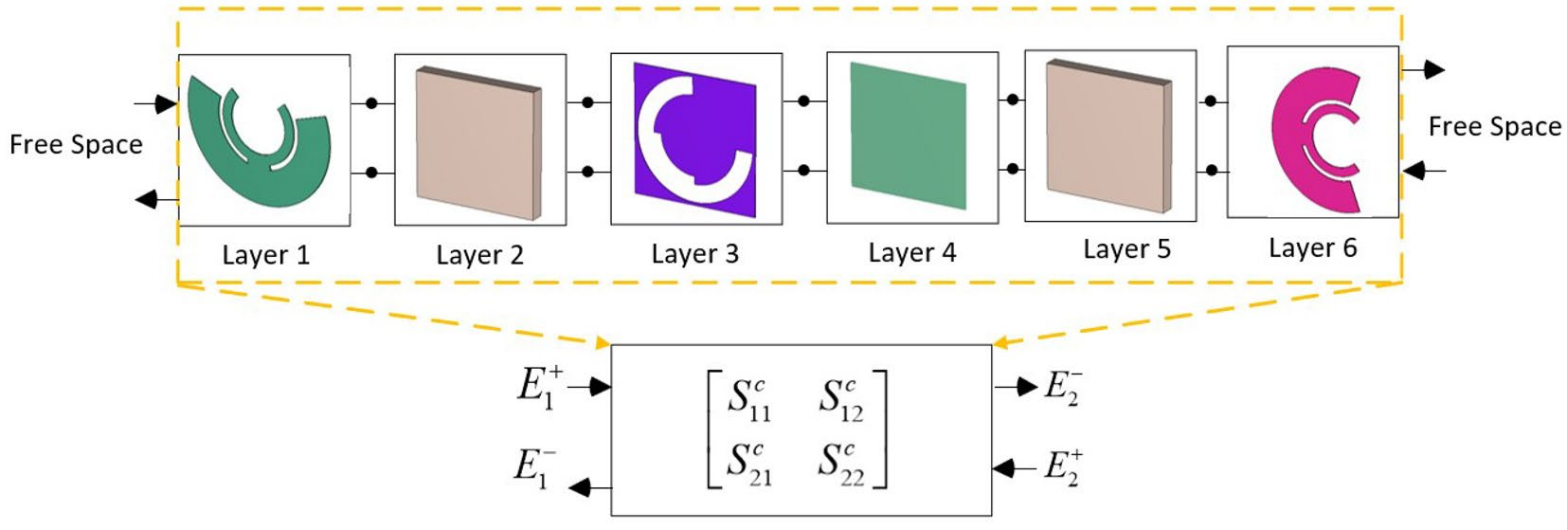
Generation of [S] matrix from six cascaded layers of the element including three conducting layers.

It was demonstrated that a quad-layer transmissive metasurface with three conductive layers can provide a full range of ${360^ \circ }$ with a transmission amplitude better than − 1 dB when identical conducting layers are employed as frequency selective surfaces (FSS)^2^. However, the phase range can be extended by using conducting layers with different metal patches. This extension is applicable within a limited bandwidth, and to achieve optimal phase and amplitude responses across a wide range of frequencies, a unit cell with multi-degree-of-freedom technology is required. The electromagnetic response of the unit cell in an array environment can be described by the scattering matrix, neglecting the higher-order mode coupling effect. As a general rule, the higher-order mode coupling effect decreases as the substrate thickness and permittivity increase. Through a cascading process, the overall scattering matrix of all six layers of the proposed phasing element can be extracted by considering each layer as a two-port system (Fig. 3). If the individual conducting layers are lossless, reciprocal, and symmetrical, the scattering matrix ([S] matrix) can only be described as a function of the transmission phase^2^. The [S] matrix for the conducting layers 1, 3 and 6, along with the [S] matrix associated with dielectric layers 2, 4, and 5, are shown in Eq. (1).

1 $$\begin{aligned} \left[ {{\text {S}}^{1,3,6}} \right]&=\left[ \begin{array}{lll} \sin \left( \measuredangle S_{21}^{1,3,6} \right) {{e}^{j\left(  \measuredangle S_{21}^{1,3,6}\pm \frac{\pi }{2} \right) }} &{}\quad \cos \left( \measuredangle S_{21}^{1,3,6} \right) {{e}^{j\left( \measuredangle S_{21}^{1,3,6} \right) }} \\ \cos \left( \measuredangle S_{21}^{1,3,6} \right) {{e}^{j\left( \measuredangle S_{21}^{1,3,6} \right) }} &{}\quad \sin \left( \measuredangle S_{21}^{1,3,6} \right) {{e}^{j\left( \measuredangle S_{21}^{1,3,6}\pm \frac{\pi }{2} \right) }} \\ \end{array} \right] \nonumber \\ \left[ {{\text {S}}^{k}} \right]&=\frac{\left( 1-{{e}^{-2j{{\beta }^{k}}L_{d}^{k}}} \right) }{1-{{\left( {{\Gamma }^{k}} \right) }^{2}}{{e}^{-2j{{\beta }^{k}}L_{d}^{k}}}}\left[ \begin{array}{lll} {{\Gamma }^{k}} &{}\quad 1-{{\left( {{\Gamma }^{k}} \right) }^{2}} \\ 1-{{\left( {{\Gamma }^{k}} \right) }^{2}} &{}\quad {{\Gamma }^{k}} \\ \end{array} \right] \end{aligned}$$

In (1), ${{\Gamma }^{k}}=\frac{1-\sqrt{\varepsilon _{r}^{k}}}{1+\sqrt{\varepsilon _{r}^{k}}}$, ${{\beta }^{k}}=\frac{2\pi }{{{\lambda }_{0}}}\sqrt{\varepsilon _{r}^{k}}$, and $k=2,4,5$ is the layer number. By cascading the [S] matrices of conducting and dielectric layers presented in (1), the overall [S] matrix will be obtained. In general, the formulation for cascading two individual layers are as follows^2^:

2 $$\begin{aligned} \left[ \begin{array}{lll} S_{11}^{c} &{}\quad S_{11}^{c} \\ S_{21}^{c} &{}\quad S_{22}^{c} \\ \end{array} \right] =\left[ \begin{array}{lll} \frac{S_{11}^{i+1}S_{12}^{i}S_{21}^{i}}{1-S_{11}^{i+1}S_{22}^{i}}+S_{11}^{i} &{}\quad \frac{S_{21}^{i}S_{21}^{i+1}}{1-S_{11}^{i+1}S_{22}^{i}} \\ \frac{S_{21}^{i}S_{21}^{i+1}}{1-S_{11}^{i+1}S_{22}^{i}} &{}\quad \frac{S_{22}^{i}S_{12}^{i+1}S_{21}^{i+1}}{1-S_{11}^{i+1}S_{22}^{i}}+S_{22}^{i+1} \\ \end{array} \right] \end{aligned}$$

In (2), the parameter $S_{21}^{c}$ represents the transmission coefficient of the cascaded layers with numbers of *i* and $i + 1$. By repeating the cascading process for all pairs consecutive layers and replacing them with the cascaded layer, the final one two-port system will be obtained which could be described in the form of ${{A}_{overall}}\cdot {{e}^{j{{P}_{overall}}}}=f\left( \measuredangle S_{21}^{1},\measuredangle S_{21}^{3},\measuredangle S_{21}^{6} \right)$ assuming the electrical thickness of the dielectric layers remains fixed.

## Unit cell simulation results

**Figure 4:**
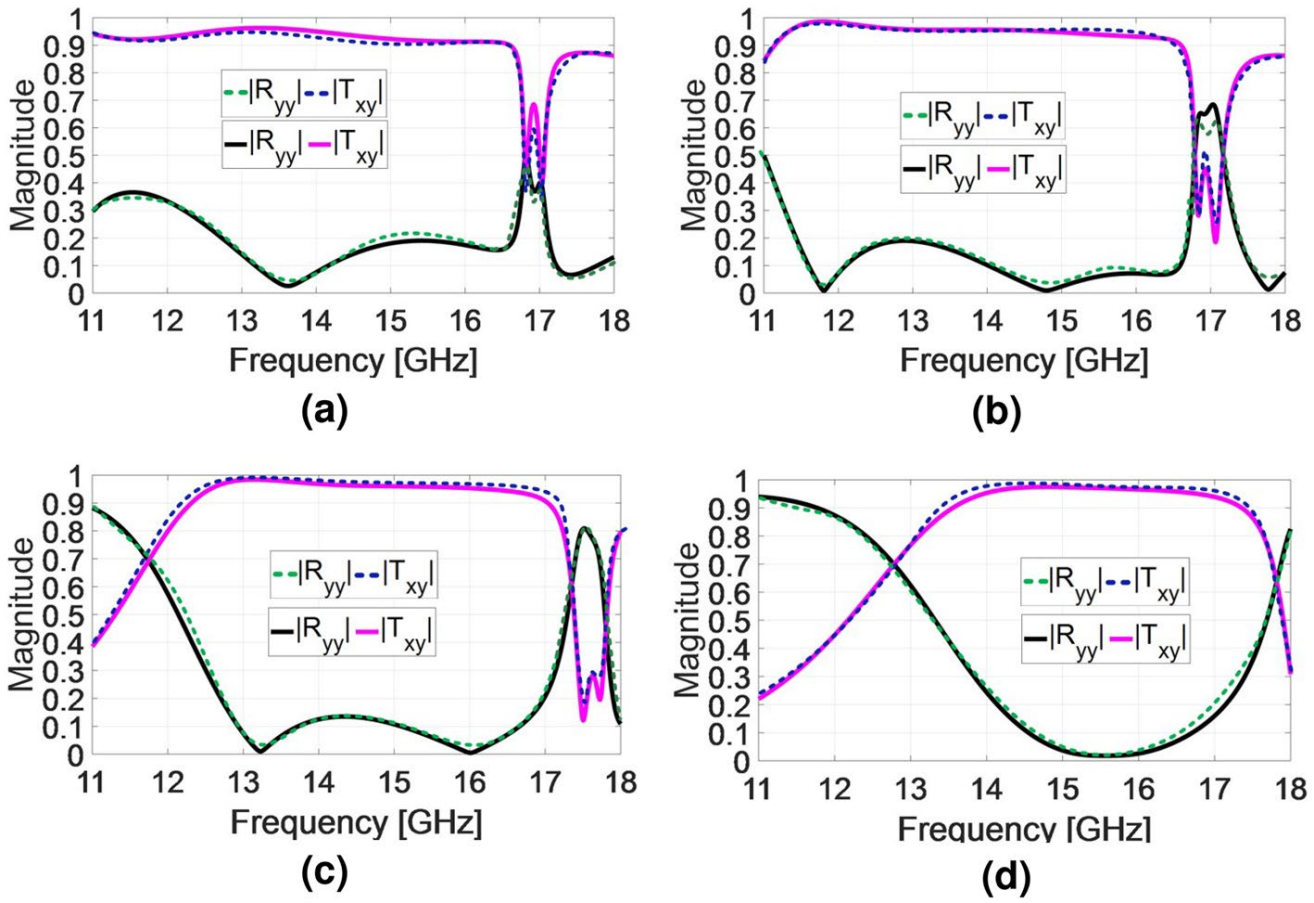
(**a**) Transmission and reflection coefficients for the y-polarized incident wave (**a**) state 1, (**b**) state 2, (**c**) state 3 (**d**) state 4. (The dashed lines show the output results of the overall cascaded [S] matrix).

**Figure 5:**
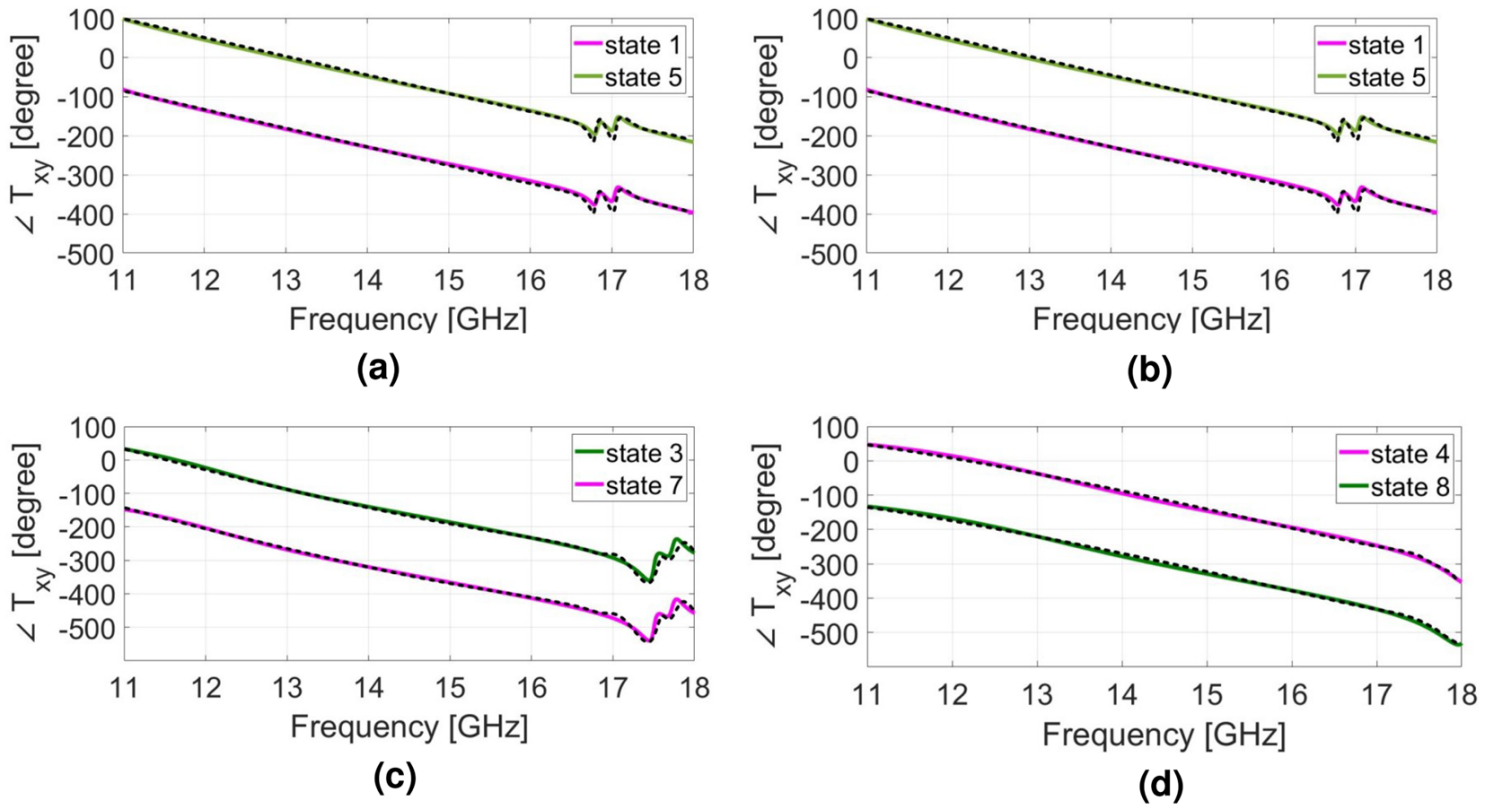
(**a**–**d**) The phase response of all 8 states from 11 to 18 GHz.

The simulated transmission and reflection amplitudes versus frequency are compared with those obtained by overall cascading [S] matrix in Fig. 4. The theoretical calculation results of the amplitude of the transmitted and reflected wave are shown by dashed lines in Fig. 4. The results demonstrate good consistency between the full-wave simulation and theoretical analysis. The transmitted phase response of twisted polarization wave for all 8 states are shown in Fig. 5 when the incident wave is y-polarized. All phase responses exhibit relatively linear behavior up to 16.5 GHz. These achievements are also verified through the proposed theoretical analysis described in the previous section. The dashed lines in Fig. 5 show the phase responses of the unit cell which are calculated by [S] matrix method. It could be seen that a good consistency exist between the simulation results and those obtained by [S] matrix method. The simulations were conducted using CST software with the periodic boundary conditions in the x- and y-directions and open space in the -z and +z-directions. It is evident that the x-polarized transmission coefficient is larger than 0.9 for states of 1,2,3, and larger than 0.56 for state of 4 within the working frequency band. It is worth nothing that the [S] matrix method is a semi-analytical approach since the quantity $\measuredangle S21$ in matrix of Eq. (1) is calculated individually for each conducting layer using CST software, and then they are cascaded using the formulation provided in Eq. (1).

**Figure 6:**
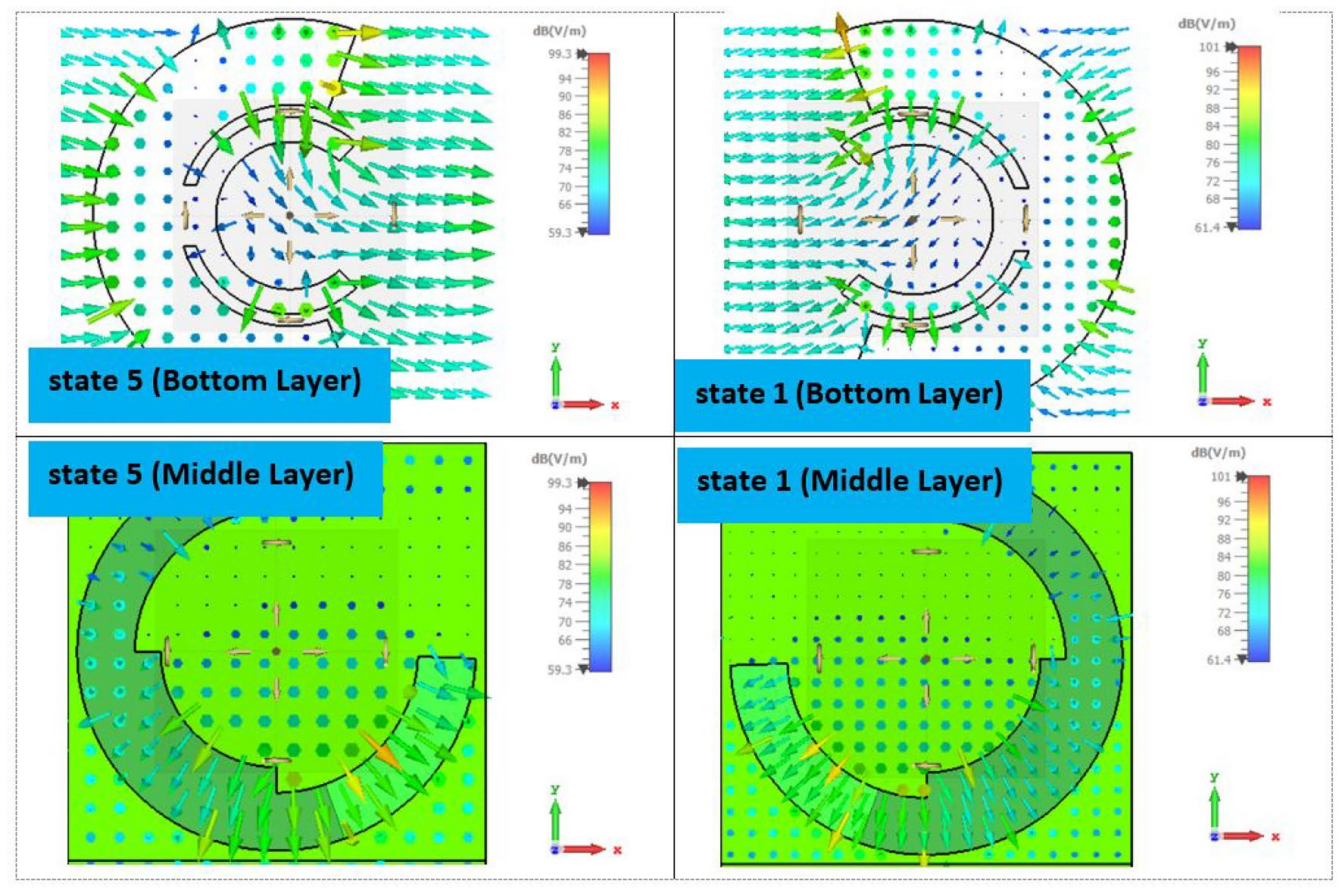
Electric field distribution on the middle and bottom layers associated with the states of 1 and 5 at 14 GHz.

**Figure 7:**
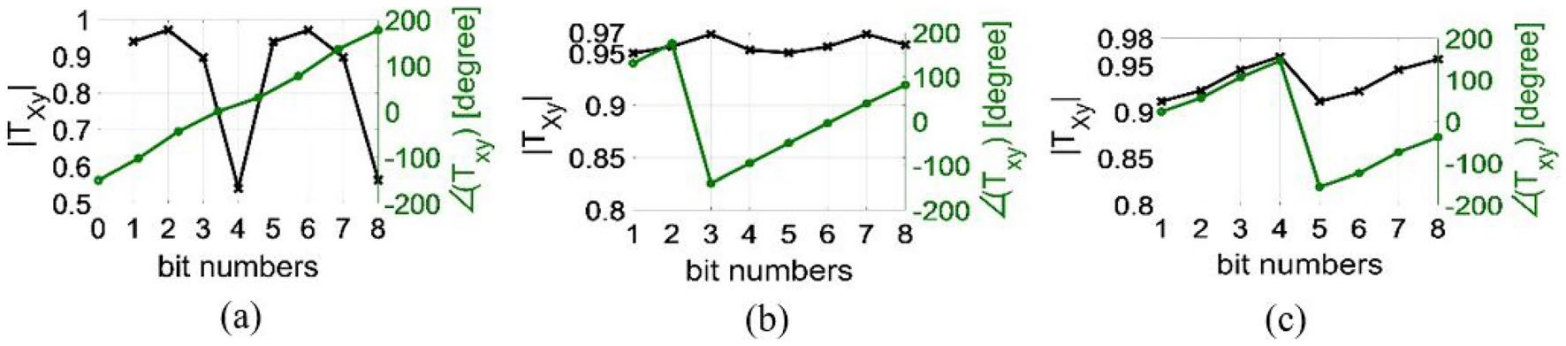
Amplitude and phase of transmission coefficients of all 8 states at (**a**) 12.3 GHz, (**b**) 14 GHz, and (**c**) 16.5 GHz.

Similar results are obtained for states of 5 to 8 although they are not shown here. To elucidate the phase behavior of the element when the middle and bottom layers are rotated to achieve states 5 to 8, a full-wave simulation is performed, and the electric field distribution over these surfaces is investigated. Figure 6 depicts the electric field distribution on the middle and bottom layers for states 1 and 5 at 14 GHz. A clear ${180^ \circ }$ phase difference between states 1 and 5 is observed from the direction of the electric fields on the bottom layers, which are opposite to each other. In summary, the amplitude and phase of the transmission wave for all 8 states are shown in Fig. 7 at the center and extreme frequencies of the band.

## MFPS approach for 3-bit TFM

The present paper employs the MFPS approach (as introduced by^24^ along with subsequent studies that aimed to design broadband RFM^3,22^) at the system design level to achieve the maximum 1-dB gain bandwidth and aperture efficiency as much as possible. The objective is to optimize the phase distribution on the aperture using this approach to balance the overall phase error caused by 3-bit phase quantization. Initially, the frequency band is set from 12.3 to 16.5 GHz, based on the amplitude and phase behavior of all bits. To achieve this, a global search algorithm known as particle swarm optimization (PSO) is utilized to balance the phase error within the frequency band and obtain an optimal phase distribution for the TFM. In the PSO algorithm, the cost function considers the sum of phase error of all elements on the aperture, along with the gain variance, to guide the optimization process.

It is important to note that the objective of using the PSO in this work is not solely to obtain the best phase distribution at a specific frequency sample in order to achieve the highest gain for that sample. Pursuing such an approach would result in maximum gain and aperture efficiency at only one frequency, leading to a reduced bandwidth. This approach is commonly referred to as the “Single frequency phase synthesis approach.” It is evident that achieving zero phase error across the entire frequency band is impossible. Instead, we propose considering the total phase error of all elements on the aperture, along with the gain variance, as the cost function in the PSO. This ensures that the gain variation is minimized across the entire band. Additionally, by designing the unit cell to minimize loss during the transmission of the wave, we can maximize the aperture efficiency. Also, several strategies have been implemented at the system design level during the implementation of MFPS to achieve optimal performance in terms of bandwidth and efficiency. These strategies are discussed below:

- Utilizing full-wave simulation results of the amplitude and phase of the horn field across the aperture.
- Incorporating full-wave simulation results for the amplitude of the transmission coefficient from the unit cell across all frequencies involved in the optimization process. This is achieved by considering the necessary phase and the corresponding state number based on Fig. 5.
- Employing a horn with a relatively stable phase center and a symmetric radiation pattern. The average of the simulated phase centers across the entire frequency band is used to determine the optimal position for the horn.
- Selecting the frequency at the beginning of the band (12.3 GHz) as the reference point in the optimization routine. This frequency is given the highest weighting factor to minimize phase errors.

In an ideal single-frequency phase synthesis approach, the gain versus frequency curve exhibits an ascending behavior. Thus, by introducing more phase errors towards the end of the band compared to the frequencies at the beginning, a flat gain response can be achieved. The required phase to transform a spherical incident wave to a planar wave focusing on $\left( {{\theta }_{b}},{{\phi }_{b}} \right)$, is determined as follows:

3 $$\begin{aligned}&\varphi _{mn}^{req}(f)={{\varvec{\varphi }}_{\textbf{0}}}(f)+ \frac{2\pi c}{f}\left( {{\varphi }_{feed}}(f)-{{x}_{mn}}\sin {{\theta }_{b}}\cos {{\phi }_{b}}-{{y}_{mn}}\sin {{\theta }_{b}}\sin {{\phi }_{b}} \right) \end{aligned}$$

In (3), ${{\varphi }_{feed}}(f)$ represents the phase of the electric field of the horn on the aperture which is extracted from CST software at the frequency *f*, ${{\varvec{\varphi }}_{\textbf{0}}}(f)$ denotes the constant reference phase at *f*. The parameters ${{x}_{mn}}$, and ${{y}_{mn}}$ indicate the position of the unit cells on the aperture plane. As stated, the cost function in MFPS approach takes into account the sum of phase errors for all unit cells, as well as the variance of the gain across all frequencies considered for optimization. The cost function is defined as follows:

4 $$\begin{aligned} COST&=\sum \limits _{{{f}_{i}}}{\left[ {{w}_{{{f}_{i}}}}\sum \limits _{m}{\sum \limits _{n}{{{M}_{mn,bit}}\left( {{f}_{i}} \right) \cdot {{P}_{mn}}({{f}_{i}})}} \right] } \nonumber \\&\quad +{\text {var}}\left[ G\left( {{f}_{i}} \right) \right] ,\,\,\,{{P}_{mn}}({{f}_{i}})=\left| \varphi _{mn}^{req}({{f}_{i}})-\varphi _{mn}^{achievable}({{f}_{i}})\, \right| \end{aligned}$$

In (4), ${{f}_{i}}$ represents 10 equispaced samples from 12.3 to 16.5 GHz, ${{P}_{mn}}$ denotes the phase error, $\varphi _{mn}^{req}\left( {{f}_{i}} \right)$ is the required phase at ${{f}_{i}}$, which is determined according to Eq. (3), $\varphi _{mn}^{achievable}\left( {{f}_{i}} \right)$ is the realizable or achievable phase at ${{f}_{i}}$ which could be provided by the unit cell, ${{w}_{{{f}_{i}}}}$ is set as a decreasing arithmetic sequence of $[1,0.9,0.8,\ldots ,0.1]$, ${\text {var}}\left[ G\left( {{f}_{i}} \right) \right]$ represents the variance of the gain values of the radiation pattern within the frequency band, computed for all particles in each iteration of the PSO algorithm. It is necessary to mention that the Fast Fourier Transform (FFT) technique is utilized to expedite the algorithm in the far-field pattern calculation step^1^. The coefficients ${{M}_{mn,bit}}\left( {{f}_{i}} \right)$ depend on the illumination intensity at ${{f}_{i}}$ which are defined as:

5 $$\begin{aligned} {{M}_{mn,bit}}\left( {{f}_{i}} \right) =\left| E_{mn}^{Horn,full-wave}\left( {{f}_{i}} \right) \right| \left| T_{mn,state}^{Element,full-wave}\left( {{f}_{i}} \right) \right| \end{aligned}$$

As can be seen in (5), these coefficients are obtained by multiplying the magnitude of the co-polarization field of the horn on the aperture, $\left| E_{mn}^{Horn,full-wave}\left( {{f}_{i}} \right) \right|$, and the magnitude of the transmission coefficient of the $m{{n}^{th}}$ element $\left( \left| T_{mn,\,state}^{Element,full-wave}\left( {{f}_{i}} \right) \right| \right)$. These two factors are extracted from CST software considering the state number of the element and the position of the element with respect to the horn antenna. Finally, the achievable phase realized by the practical coding surface is defined as follows:

6 $$\begin{aligned}&\varphi _{mn}^{achievable}={{\varphi }_{bit}}\,\, \nonumber \\&\quad \text {if } \left| {{\varphi }_{bit}}-\varphi _{mn}^{req} \right| <\left| \varphi _{bit}^{o.w}-\varphi _{mn}^{req} \right| ,\,\,\,\,bit=1,2,\ldots ,8 \end{aligned}$$

In (6), ${{\varphi }_{bit}}$ represents one of 8 states defined in Fig. 2 and $\varphi _{bit}^{o.w}$ refers the phases other than ${{\varphi }_{bit}}$ according to Fig. 2.

To demonstrate the feasibility of the proposed combined broadband technique, a proof-of-concept 25 $\times$ 25-element TFM is designed in Ku-band. A Ku-band tangential profiled smooth-wall horn with $q=6.5$, which was employed in our previous work^3^, is chosen as the feeding source. The number of particles and iterations are considered 250 and 1000, respectively, to ensure convergence. The final optimum constant reference phases are obtained as $[-26, -80, 67, 114, -177, -158, -47, -26, -78, 81]$ degrees corresponding to 10 frequency samples.

**Figure 8:**
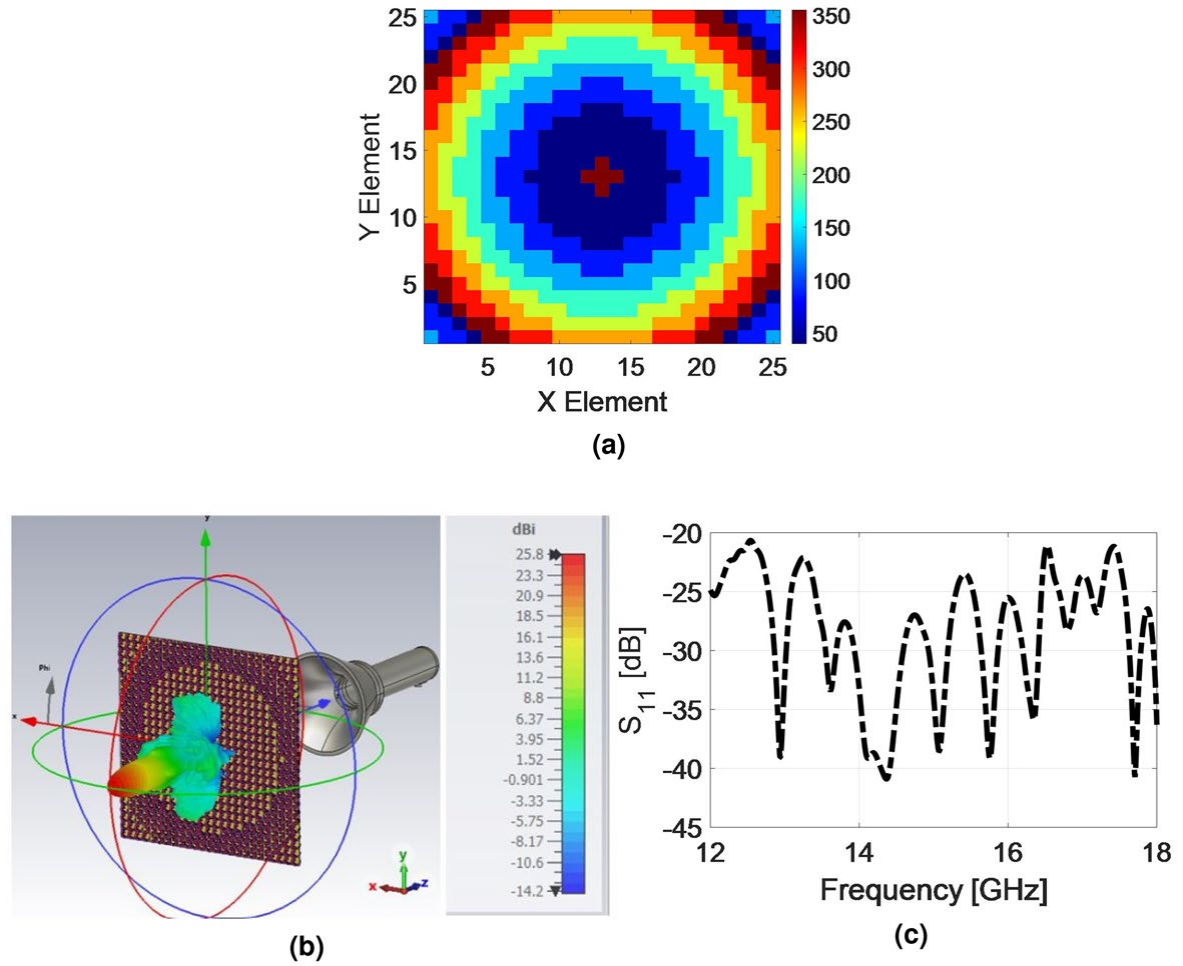
(**a**) Optimal phase distribution at 14 GHz to achieve a broadband high-efficiency TFM. (**b**) 3D demonstration of absolute gain of the designed TFM at 14 GHz. (**c**) Simulated S11 of TFM.

## Results and discussion

Based on the criteria outlined in the previous section, the optimal phase distribution of the TFM is obtained and illustrated in Fig. 8a. The main beam direction is considered as $\left( {{\theta }_{b}},{{\varphi }_{b}} \right) =\left( {{0}^{\circ }},{{0}^{\circ }} \right)$. By mapping the 8 phase states of the element represented in Fig. 2 to the phase distribution of Fig. 8a, the final arrangement of the elements in array environment is obtained for all three layers. Subsequently, the designed TFM and the previously described feeding source (a smooth-wall conical horn antenna) are simulated using the time domain solver of the CST software. The simulations are performed on a computer system with 64 GB of RAM. The feeding source is positioned in front of the TFM, taking into account the average phase center value relative to the frequency range of operation. The simulation of the designed TFM, with dimensions of 16.5 mm $\times$ 165.5 mm, takes approximately 8 h to complete. The simulation results, including the S11 parameter and the 3D radiation pattern of the TFM at 14 GHz, are presented in Fig. 8b,c, respectively. The simulated 3D pattern demonstrates the satisfactory performance of the designed TFM in focusing the emitted waves from the horn. The peak gain achieved at 14 GHz is 25.8 dBi, corresponding to an aperture efficiency of 52.6%. Moreover, the S11 of the structure defined from the input port of the horn as the feeding source remains below − 20 dB which is considered acceptable. This indicates that the majority of the wave energy passes through the TFM, aligning with the predictions derived from the simulation results presented in Fig. 4.

**Figure 9:**
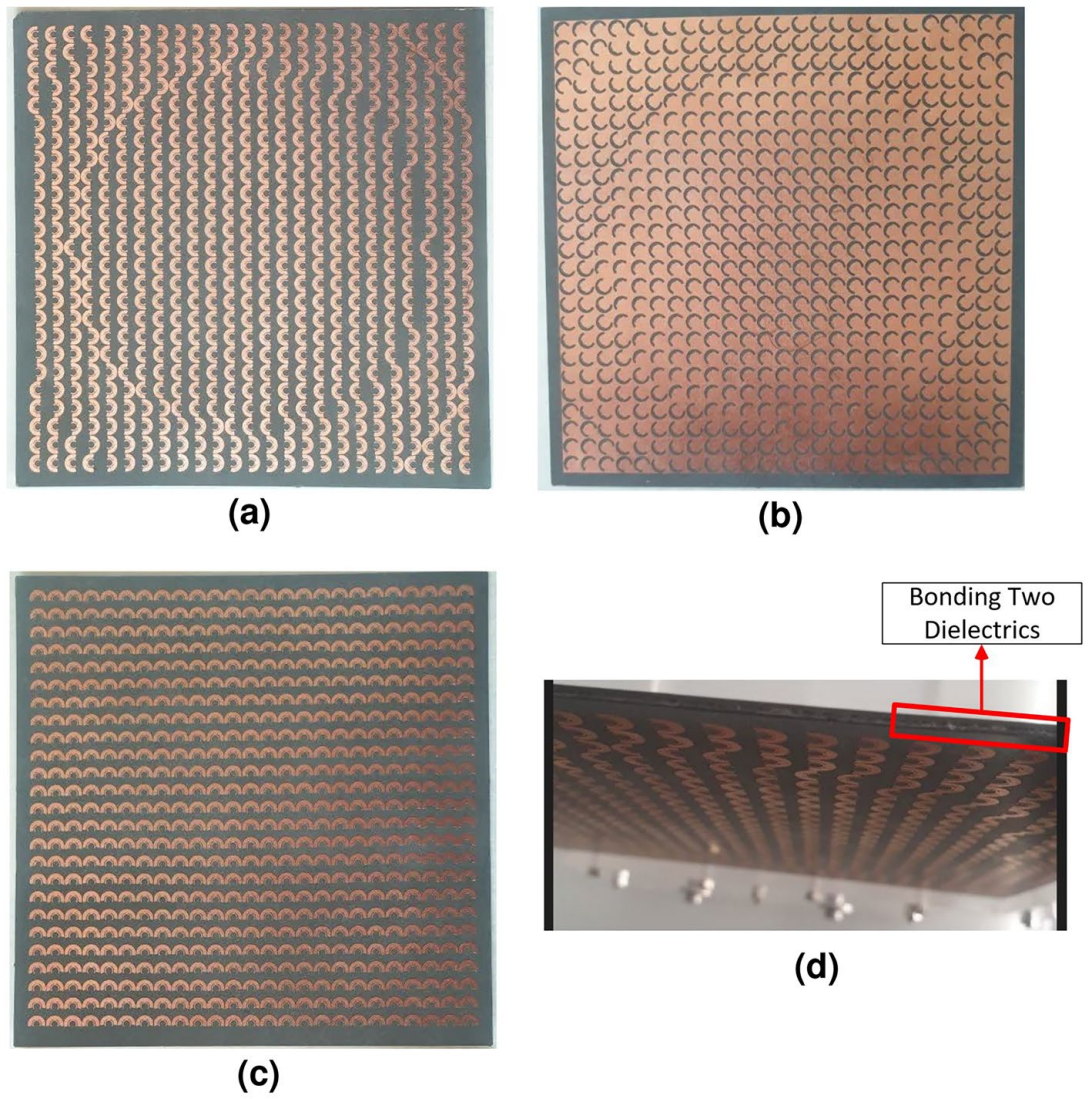
Fabricated two layers of TFM. (**a**) Front view of the top layer. (**b**) Back view of the top layer. (**c**) Front view of the bottom layer. (**d**) Close view from the bonding region of the two dielectrics.

**Figure 10:**
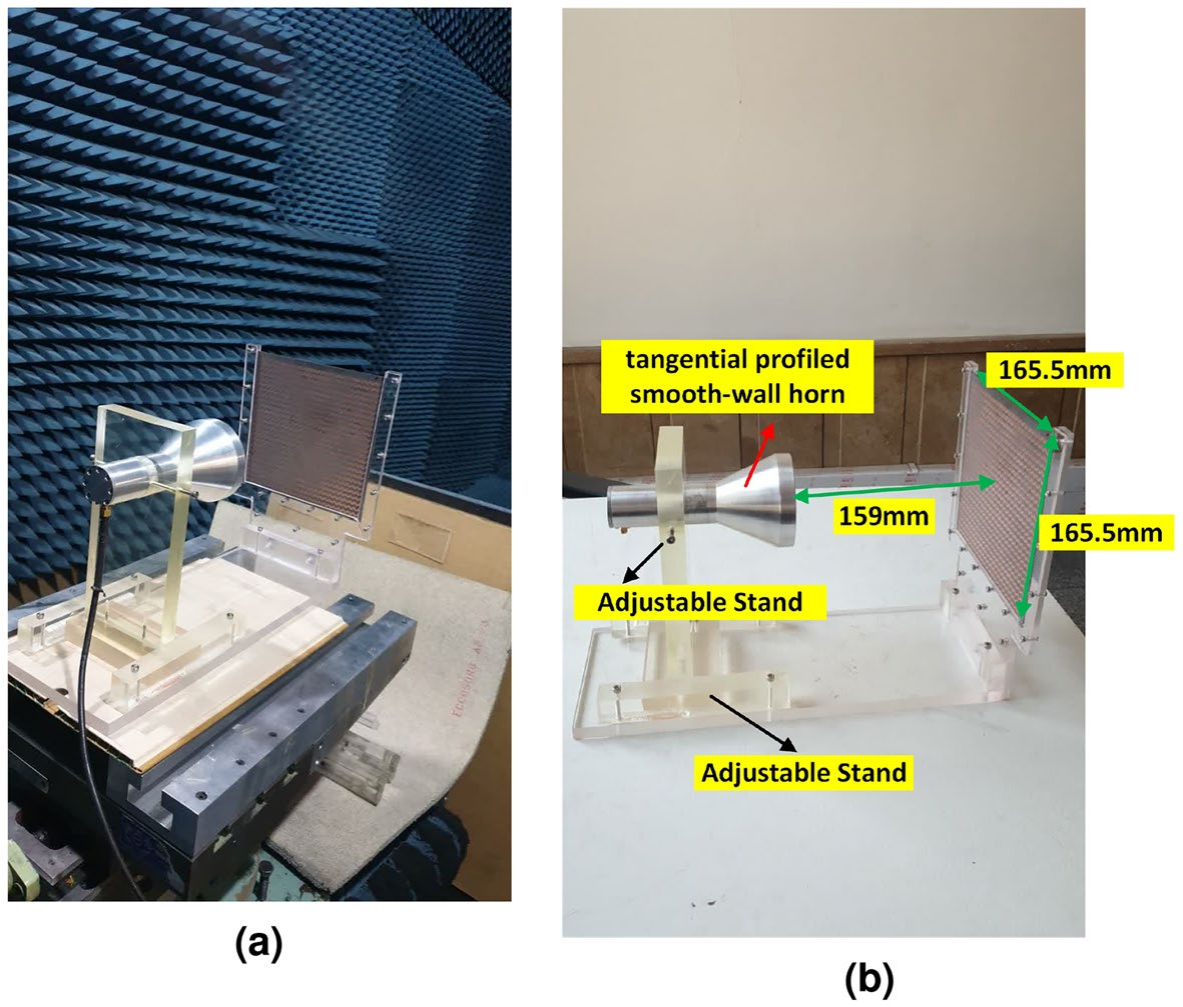
(**a**) Designed metasurface and its adjustable stand to hold the feed. (**b**) Measurement setup in an anechoic chamber.

**Figure 11:**
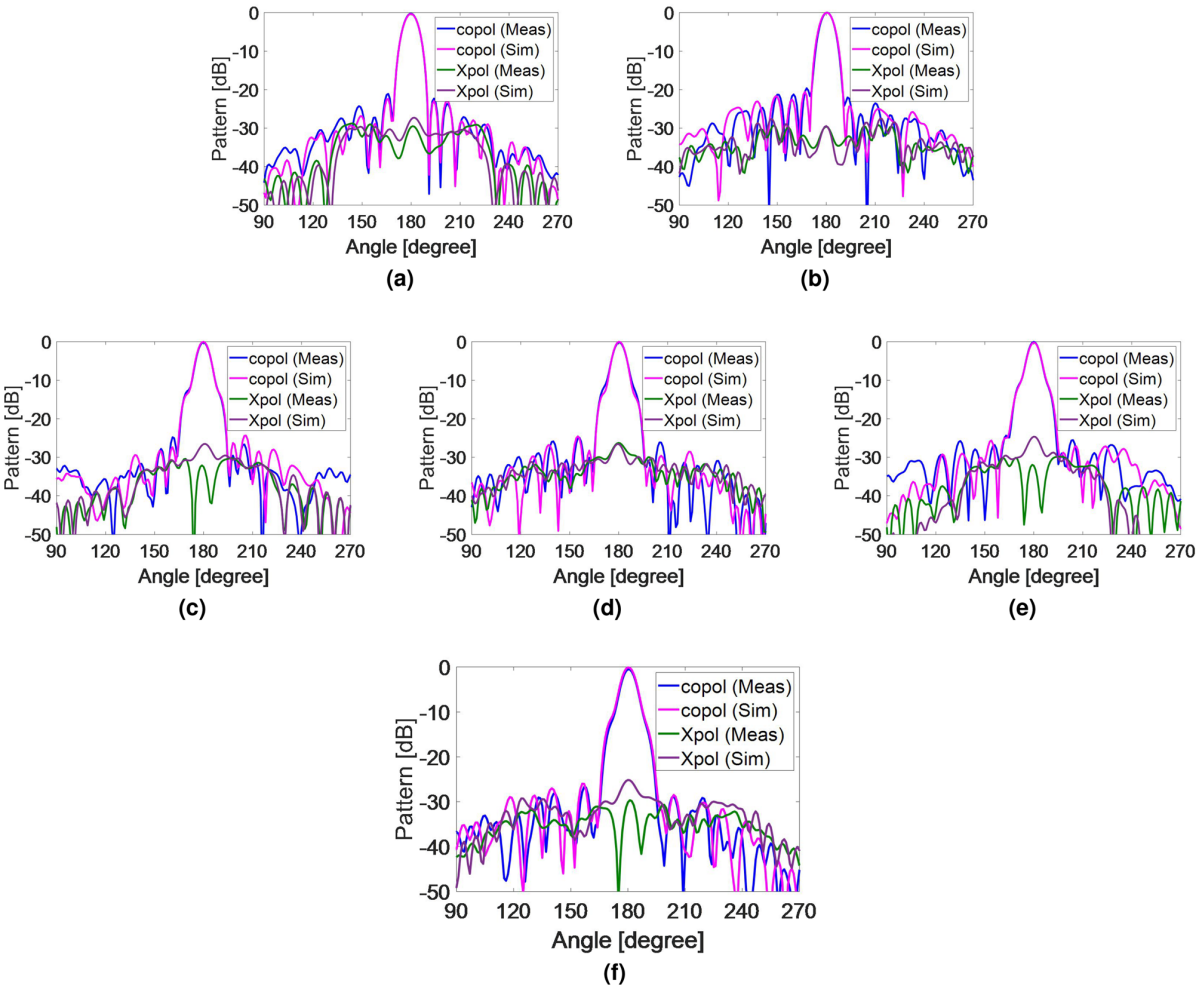
Simulation and measurement results of the normalized gain in the E- and H-planes (**a**) E-plane at 12.3 GHz, (**b**) H-plane at 12.3 GHz, (**c**) E-plane at 14 GHz, (**d**) H-plane at 14 GHz, (**e**) E-plane at 16.5 GHz, (**f**) H-plane at 16.5 GHz.

**Figure 12:**
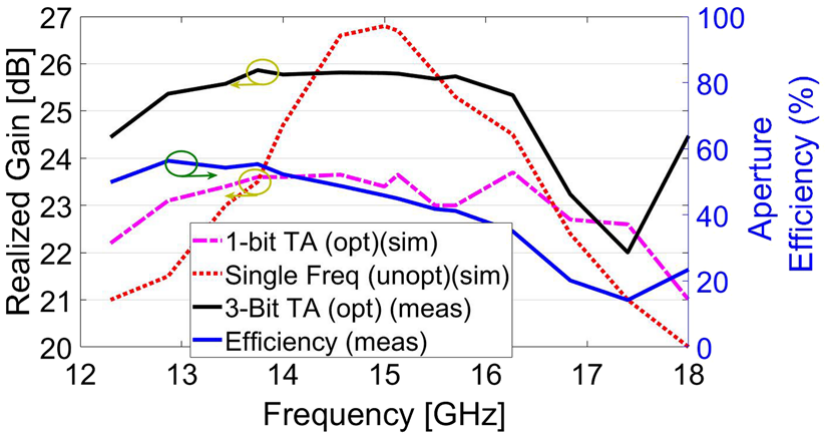
Comparison between different scenarios to design a TFM along with the measured aperture efficiency. “opt” and “unopt” stands for the optimized and unoptimized model, respectively.

To experimentally validate the designed broadband high-efficiency TFM, a prototype was fabricated using printed circuit board (PCB) technology. The fabricated metasurface and measurement setup are illustrated in Figs. 9 and 10. In order to fix the position of the feed relative to the TFM, a setup made of plexiglass with a flexible focal length was utilized with. The TFM was enclosed by a sturdy frame and secured with plastic screws. The far-field radiation patterns are measured at the center and extreme frequencies of the band in an anechoic chamber. Figure 11 compares the simulated and measured far-field patterns in the E- and H-planes, demonstrating good consistency between them. The measurement results indicate that the side lobe levels remain below − 20 dB for all three frequency points in both planes and the cross-polarization component of the pattern remain below − 28 dB at all angles. It is worth mentioning that the designed unit cell element has a similar electromagnetic behavior when the wave is illuminated to the aperture in the opposite direction (-z-direction) with orthogonal polarization. Therefore, if the horn is positioned on another side of the TA with the same *f*/*D*, and rotated by ${{90}^{\circ }}$ around its longitudinal axis, it is expected that the transmitted wave would exhibit similar characteristics as the results shown in Fig. 11. The measured 1-dB gain bandwidth and the corresponding aperture efficiency of the proposed metasurface are depicted in Fig. 12. The results reveal a maximum realized gain of 25.8 dB and a maximum aperture efficiency measured at 12.8 GHz, which corresponds to 53.6%. To highlight the advantage of using the MFPS approach integrated with multi-bit coding metasurface concept to simultaneously improve the bandwidth and efficiency of TFM, two additional TFMs were designed. The first one follows the classic single-frequency phase synthesis approach, in which the phase error is minimized at a single frequency referred to as the *design frequency*. The second one is a single-bit TFM with two states for the phase distribution, (state of 3 and 6 in Fig. 2), in which the MFPS approach is also employed. The Simulation results comparing the peak gain versus frequency for all three configurations are presented in Fig. 12.

The comparison of the peak gains shows that the use of single-bit TFM has a wider 1-dB bandwidth from 12.7 GHz to 17.5 GHz compared to the other two radiators. However, the maximum gain of this metasurface is nearly 2 dB lower than the 3-bit configuration which in turn causes to lower aperture efficiency.

Additionally, the side lobe levels in single-bit coding TFM are obtained at approximately − 17 dB for all frequencies of optimizations, which are higher than side lobe levels in 3-bit TFM. This difference can be attributed to the fact that the phase distribution in multi-bit configurations is closer to the ideal phase distribution dictated by the ray tracing theory in space-fed metasurfaces. In the second simulation, the design frequency is set to 15 GHz. As shown in Fig. 12, it is evident that the minimum phase error occurs at 15 GHz. The maximum peak gain of singe-frequency TFM is obtained to be 26.9 dB, which is approximately 1.2 dB higher than the 3-bit TFM. This demonstrates that in the MFPS approach, the phase error is balanced rather than minimized within the frequency band to achieve a smoother gain response. A comparison is conducted between the performance of the presented work and the previously published studies which are summarized in Table 2. The comparison highlights that the presented TFM achieves both high aperture efficiency and a wide 1-dB gain bandwidth by leveraging the combined broadband technique integrated with multi-bit coding metasurface.

**Table 2:** Comparison of the proposed 3-bit wideband high-efficiency TFM with other works.

Ref. number	This work	Ref.^25^	Ref.^26^	Ref.^27^	Ref.^28^	Ref.^29^
Freq (GHz)	14	10	15	27	8.5	150
Via	No	No	No	Yes	Yes	No
Technology	PCB	PCB	PCB	PCB	Metal Only	PCB
Phase. Dist	3-Bit	2-Bit	Continuous	1-Bit	1-Bit	six states
Dielectric. Num	2	2	3	4	2	2
Max. AE	53.4%	17%	42.3 %	28 %	47.6 %	32 %
1-dB Gain	%29	33.4 % (3-dB)	37.3 %	N. A	14.6 %	16.8 %
Fabrication	Easy	Easy	Easy	Difficult	Difficult	Easy
Thickness	0.14${{\lambda }_{0}}$	0.05${{\lambda }_{0}}$	0.165${{\lambda }_{0}}$	0.16${{\lambda }_{0}}$	0.23${{\lambda }_{0}}$	0.25${{\lambda }_{0}}$

## Conclusion

This paper presents the design, fabrication, and testing of a novel broadband high-efficiency coding metasurface operating in transmissive mode. The integration of a multi-bit configuration and the MFPS approach allows for minimizing the phase error within the frequency band, resulting in a flat gain response. The main objective is to reduce phase errors at the lower frequencies of the band, considering the ascending gain curve versus frequency. This approach enables the achievement of a gentle gain response, with more phase errors forced at the higher frequencies towards the end of the band. The designed metasurface exhibits a 1-dB gain bandwidth of 29% and a maximum aperture efficiency of 53.6%.

## Data Availability

All data required to evaluate the findings of this work is available in the presented paper. Additional data related to this work may be requested from the corresponding author.
